# Transplanted Human Induced Pluripotent Stem Cell-Derived Neurons Wire and Fire with Balanced Excitation–Inhibition in Rat Cortex

**DOI:** 10.1523/ENEURO.0041-20.2020

**Published:** 2020-04-13

**Authors:** Rosalind S.E. Carney

## Abstract

**Highlighted Research Paper:** Neurons Derived from Human Induced Pluripotent Stem Cells Integrate into Rat Brain Circuits and Maintain Both Excitatory and Inhibitory Synaptic Activities. Xiling Yin, Jin-Chong Xu, Gun-sik Cho, Chulan Kwon, Ted M. Dawson and Valina L. Dawson.

The ability to induce pluripotent stem cells (PSCs) to generate cortical neurons is a foundation of cell-based therapies for neurological disorders that involve neuronal loss or dysfunction. Cell-based therapies aim to replace lost/damaged cell populations or to replenish sources of growth factors (Alia et al., 2019). More than a decade ago, it was shown that stimulation of embryonic stem cells (ESCs) with exogenous patterning factors could generate diverse phenotypes of cortical neurons capable of structural and functional integration into recipient mouse cortex (Gaspard et al., 2008; Eiraku et al., 2008). Candidate patterning factors to induce specific neuronal phenotypes were identified from rodent *in vivo* gene expression and genetic deletion studies that showed that the majority of excitatory (glutamatergic) projection and inhibitory (GABAergic) neurons arise from the dorsal and ventral telencephalon, respectively (de Carlos et al., 1996; Anderson et al., 1997; Tamamaki et al., 1997; Lavdas et al., 1999; Letinic et al., 2002; Nery et al., 2002). To mitigate symptoms of human neurological disorders with cell-based therapies, many research groups focus on *in vitro* model systems that can produce specific or multiple neuronal subtypes. Whereas ethical considerations preclude the use of human ESCs to generate neurons in culture (de Wert and Mummery, 2003), induced PSCs (iPSCs) are differentiated cells that have been manipulated into an ESC-like state (Takahashi and Yamanaka, 2006). Human iPSCs (hiPSCs) can be created from somatic cells in adults (Takahashi et al., 2007), providing a means to test drug efficacy and toxicity *in vitro* and the potential for personalized medicine (Shi et al., 2017). Many research groups have successfully developed different protocols to generate populations of excitatory or inhibitory interneurons in culture (Paşca et al., 2011; Shi et al., 2012; Liu et al., 2013; Maroof et al., 2013; Nicholas et al., 2013; Espuny-Camacho et al., 2017). An important challenge of cell-based therapies is to ensure that transplanted hiPSC-derived neurons survive and integrate into the recipient cortex, which has been demonstrated in several studies (Weick et al., 2011; Nicholas et al., 2013; Espuny-Camacho et al., 2017).

In a prior study, Professor Valina Dawson’s laboratory (Johns Hopkins University, Baltimore, MD) developed a culture system that generates a balanced network of excitatory and inhibitory neurons, reminiscent of the human cortex (Xu et al., 2016). It is estimated that in primate, GABAergic neurons constitute ∼25% of cortical neurons compared with ∼15% in rodents (Hendry et al., 1987; Beaulieu, 1993). The timed administration of various exogenous factors to hiPSCs permits the *in vivo* generation of GABAergic neurons and the sequential generation of deeper, then upper, layer excitatory neurons of the six-layered cerebral cortex common to rodents and humans. Neural rosettes are a symmetrically dividing population of undifferentiated neuroepithelial cells (Elkabetz et al., 2008). *In vitro*, cell aggregates derived from rosette type neural stem cells (RONAs) can be easily manipulated due to their visually distinct morphology. Xu et al. (2016) found that timed application of retinoic acid to human neural precursor cells (hNPCs) that express forebrain forkhead box G1 (FOXG1) produced a balanced excitatory and inhibitory neuronal network. The balance of neurons mimicked endogenous cortical neurons phenotypically, in terms of neurochemical expression, and functionally, in terms of electrical properties. However, it was not known whether these hiPSC-derived neurons would survive and integrate into the existing neural network of the recipient cortex following xenotransplantation. In their *eNeuro* publication, Yin et al., 2019 describe the *in vivo* fate of hiPSC-derived neurons after transplantation into the neonatal rat brain.

The authors’ original hiPSC cell line was modified to express a red fluorescent protein (RFP) to track the fate of transplanted cells. Figure 1 shows the timeline during which cultured cells developed into embryoid bodies (day 7), RONAs (day 15), neurospheres (day 29), and hNPCs (day 30). From day 30, hiPSC-derived neural progenitors were either maintained *in vitro* or dissociated before transplantation into the brain of rat pups at postnatal day 1. Each recipient (six male, six female) was injected with 200,000 cells in the right cortex; nude rats were used to avoid an immune challenge. Ten weeks after transplantation, the fate and function of hiPSC-derived neurons *in vivo* were compared with hiPSC-derived neurons maintained *in vitro* for the same duration.

**Figure 1.**
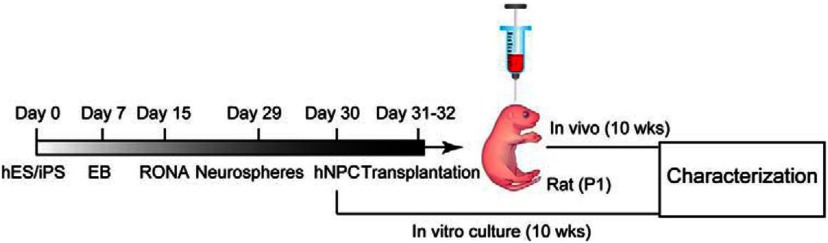
Generation and transplantation of forebrain progenitors from hiPSCs. In culture, human ESCs and induced pluripotent cells (hES/iPS) developed into embryoid bodies (EB; day 7), RONAs (day 15), neurospheres (day 29), and hNPCs (day 30). Thereafter, hNPCs were transplanted into neonatal rat cortex or maintained *in vitro*. Ten weeks after transplantation, the fate of xenotransplanted and cultured cells were compared. (Adapted from Figure 1 in Yin et al., 2019.)

After 10 weeks *in vitro*, RFP-positive (RFP+) hiPSC-derived neurons coexpressed markers representative of one or more cortical layers, including TBR1, CTIP2, BRN2, and SATB2. Electrophysiological recordings also showed that cultured RFP+ neurons displayed both spontaneous excitatory postsynaptic currents (sEPSCs) and spontaneous inhibitory postsynaptic currents (sIPSCs). These observations are consistent with the authors’ prior study (Xu et al., 2016) and demonstrate that modification of the hiPSC line did not alter cell characteristics.

Ten weeks after transplantation, immunofluorescence analysis of forebrain slices revealed that >90% of RFP+ neurons had survived *in vivo* and formed axon-like structures (Fig. 2). Patch-clamp recordings performed in *ex vivo* forebrain slices 10 weeks after transplantation showed that RFP+ neurons exhibited repetitive action potentials following depolarizing current injection. The RFP+ neurons also displayed characteristic membrane voltage changes in response to the sodium channel blocker tetrodotoxin (TTX) and tetraethylammonium (TEA), a potassium channel blocker (Fig. 3). Excitatory AMPA-receptor mediated currents were detected in RFP+ neurons when adjacent host cells were stimulated, demonstrating that RFP+ neurons functionally responded to synaptic input from the host neural network. The AMPA receptor antagonist 6-cyano-7-nitroquinoxaline-2,3-dione (CNQX) and NMDA receptor antagonist d(−)−2-amino-5-phosphonopentanoic acid (d-AP5) were also used for selective inhibition of receptor activity. Together, these observations show that hiPSC-derived neurons can functionally integrate into recipient rat cortex and display aspects of synaptic transmission characteristic of both excitatory and inhibitory neurons.

**Figure 2.**
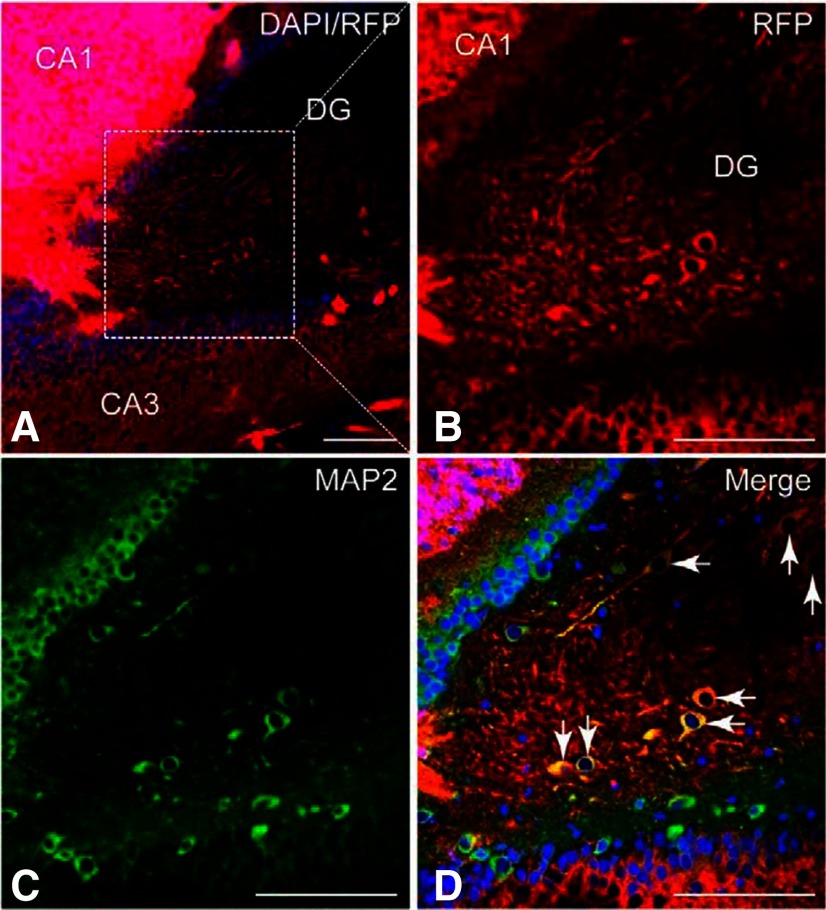
hiPSC-derived neurons integrate into the rat brain. ***A***, Representative hippocampal brain slice showing RFP+ cells (red) and nuclear DAPI stain (blue). ***B*–*D***, High-power images of the dentate gyrus (DG) from the boxed area in A showing RFP-labeled cells (***B***), immunoreactivity for the mature neuronal marker MAP2 (***C***; green), and the merged image of ***A***, ***C*** (***D***). CA, cornu ammonis; scale bar, 100 μm. (Adapted from Figure 4 in Yin et al., 2019.)

**Figure 3.**
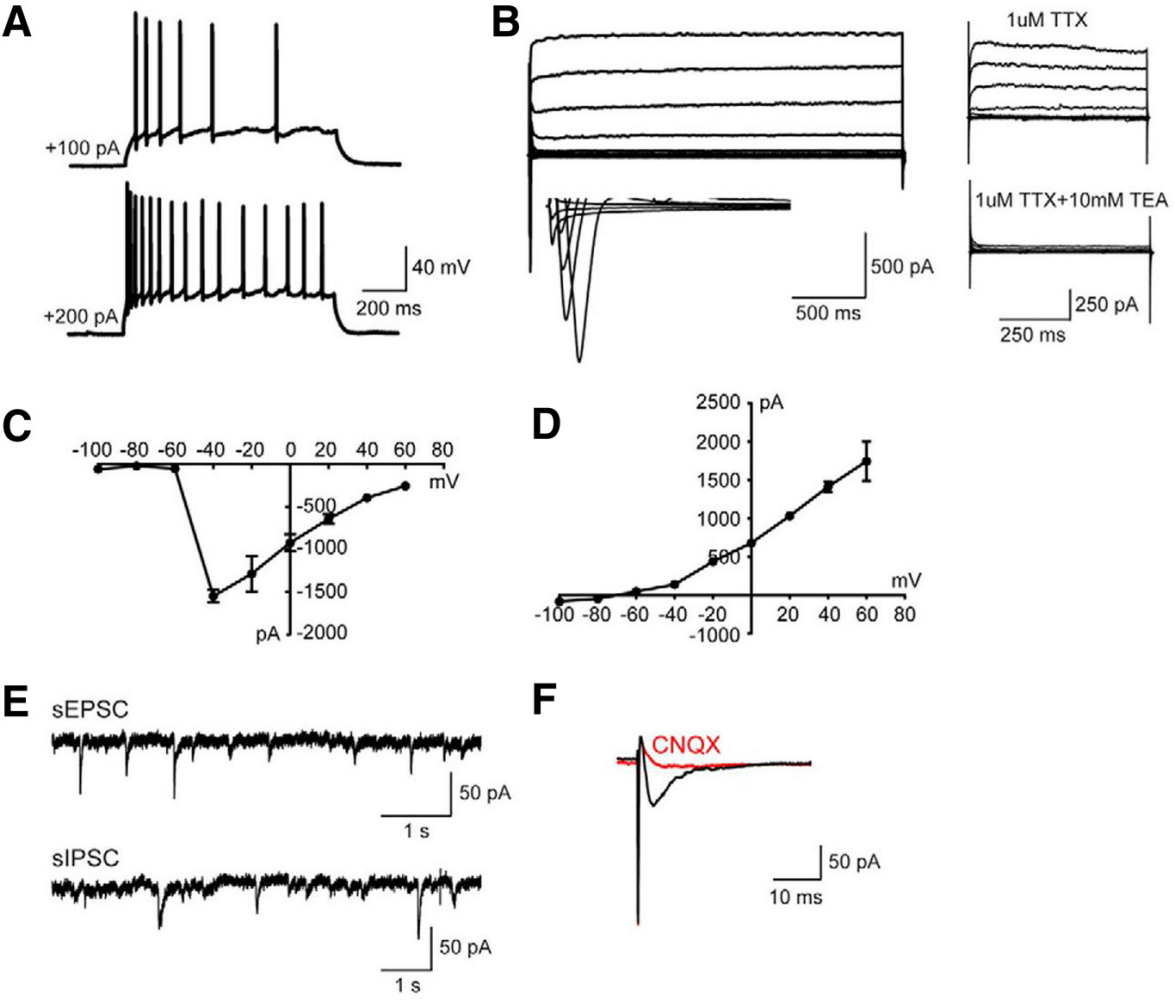
hiPSC-derived neurons functionally integrate into the synaptic circuitry of the rat brain 10 weeks after transplantation. ***A***, AP firing patterns of hiPSC-derived neurons. Increased AP firing was observed with increasing current injection. ***B***, Representative traces of whole-cell Na+ (inward) and K+ (outward) currents recorded from grafted cells, elicited by voltage steps from −100 to +60 mV in 20-mV increments, and blocked by TTX and TEA, respectively. ***C***, ***D***, I–V curves for voltage-gated Na+ (***C***) and K+ (***D***) currents. ***E***, Representative traces of sEPSCs and sIPSCs recorded in voltage-clamp configuration at −70 mV. sEPSCs were obtained in the presence of picrotoxin (100 μM), and sIPSCs were recorded in the presence of CNQX (20 μM) and d-AP5 (50 Mm). ***F***, AMPA receptor-mediated postsynaptic currents were evoked by stimulation delivered from an electrode placed ∼200–300 μm away from the transplanted cell, which was blocked by the subsequent application of CNQX. (Adapted from Figure 5 in Yin et al., 2019.)

Transplanted hiPSC-derived neurons took longer to achieve functional maturation compared with neurons maintained *in vitro*. This delay was demonstrated by whole-cell patch-clamp recordings of input resistance, cell capacitance, AP threshold, and AP width. It is, however, not surprising that integration into a host brain may impede maturation, as previously suggested (Thompson and Björklund, 2015). Lags in neuronal maturation will be an important consideration for future experimental design. Nonetheless, the current protocol permits long-term follow-up of transplanted neurons, avoiding the need for prolonged manipulation *in vitro*. In addition, transplantation provides the local host environment in which extrinsic factors may contribute to neuronal survival, synaptic transmission, and disease states.

The heterochrony of corticogenesis in rodents versus primates is an important consideration for human/rodent chimeric models. Compared with mice, the human brain has a 1000-fold difference in cortical neuron number despite a disproportionate 10-fold difference in the timing of neuron generation (Dehay et al., 2015). Comparative anatomy has revealed that “human-like” cortical features are associated with upper, rather than deep, layer neurons (Betizeau and Dehay, 2016). An enhanced outer subventricular zone (OSVZ) underlies the increase in cortical volume in primates (Smart et al., 2002; Zecevic et al., 2005; Fietz et al., 2010; Hansen et al., 2010). Particular attention needs to be paid to the xenotransplantation of OSVZ progenitor cells. Xu and colleagues’ (2016) 2-D culture system may be more appropriate than 3-D (organoid) models in this respect as the number of transplanted progenitors can be tightly controlled. Even overlooking current ethical concerns regarding xenotransplantation of organoids, the potential increase in transplanted OSVZ progenitors could generate a higher number of upper layer cortical neurons than the rodent skull could accommodate (Chen et al., 2019). Nonetheless, organoids are uniquely beneficial for *in vitro* studies as they provide tissue architecture that is lacking in 2-D culture systems (Wu et al., 2019).

The RONA culture model will also permit aspects of human corticogenesis to be further explored as postmortem human tissue samples may not be suitable if they exhibit disease states. The timed administration of other exogenous factors may lead to accelerated maturation of hiPSC-derived neurons *in vitro*. This improvement would help mitigate cost and time impediments as the prolonged maturation of cortical neurons from hiPSCs is an obstacle for the extensive use of many culture systems (Anderson and Vanderhaeghen, 2014). Because of the morphological characteristics of RONAs, this culture protocol may be adoptable by other research groups, as is, more easily than other 2-D culture protocols.

Current research in Professor Dawson’s laboratory focuses on further characterization of transplanted hiPSC-derived neurons, for example, specific neuronal subtype expression. The ultimate goal is to apply this *in vitro* differentiation protocol to neurological disorders such as stroke, Alzheimer’s disease, and Parkinson’s disease. The balanced network of excitatory and inhibitory neurons is particularly appropriate for brain regions with focal trauma, such as non-diffuse cases of stroke. Human/rodent chimeric models can determine the fate of healthy or diseased state human neurons when transplanted into rodents models of neurological disorders or wild-type rodents, respectively. Chimeric models are most suitable for monogenic disorders in which neuron-intrinsic versus extrinsic factors can be more easily distinguished than for polygenic disorders. Chimeric models using older rodents can also provide the local environment of the mature brain that is more relevant to some disorders. Age is an important consideration because to increase the translatability of results, hiPSC-derived neurons from individuals with Alzheimer’s disease and Parkinson’s disease may require aspects of aging to be mimicked *in vitro* (van den Ameele et al., 2014). When healthy iPSC-derived neurons were transplanted into a mouse model of Alzheimer’s disease, the human neurons displayed neurodegenerative changes and underwent cell death (Espuny-Camacho et al., 2017). Such observations are crucial to determine whether the focus of potential treatment strategies should lie on the neurons themselves or the environmental milieu.

Undoubtedly, the principal advance by Professor Dawson’s laboratory is the development of the RONA culture method that generates a balanced network of neurons that is representative of the ratio of excitation and inhibition in the human brain. Future research may uncover novel information about human neurological disorders that are poorly replicated in mouse models. The high failure of clinical trials investigating potential treatment options for Alzheimer’s disease is one example. The significance of hiPSC lines continues to increase, as evidenced by the recent creation of hiPSC lines for other neurological disorders such as bipolar disorder and schizophrenia (Ishii et al., 2019).
